# 4,4′-Dibromo-2,2′-{[(3a*S*,7a*S*)-2,3,3a,4,5,6,7,7a-octa­hydro-1*H*-1,3-benzimidazole-1,3-di­yl]bis­(methyl­idene)}diphenol

**DOI:** 10.1107/S1600536811006489

**Published:** 2011-03-02

**Authors:** Augusto Rivera, Diego Quiroga, Jaime Ríos-Motta, Michal Dušek, Karla Fejfarová

**Affiliations:** a Departamento de Química, Universidad Nacional de Colombia, Bogotá, AA 14490, Colombia; b Institute of Physics ASCR, v.v.i., Na Slovance 2, 182 21 Praha 8, Czech Republic

## Abstract

The cyclo­hexane ring in the title compound, C_21_H_24_Br_2_N_2_O_2_, adopts a chair conformation and the five-membered ring to which it is fused has a twisted envelope conformation. The asymmetric unit contains one half-mol­ecule, which is related to the other half by a twofold rotation axis. The two N atoms of the five-membered ring are linked to the hy­droxy groups by intra­molecular O—H⋯N hydrogen bonds. In the crystal, inter­molecular C—H⋯O and C—H⋯π inter­actions occur.

## Related literature

For a related structure, see: Rivera *et al.* (2010 ▶). For uses of di-Mannich bases, see: Mitra *et al.* (2006 ▶).
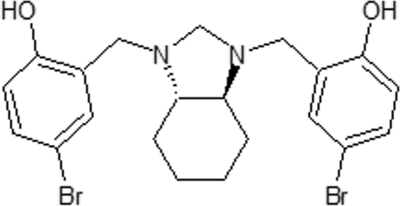


## Experimental

### 

#### Crystal data

C_21_H_24_Br_2_N_2_O_2_
*M* *_r_* = 496.2
Orthorhombic, 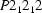

*a* = 5.9645 (2) Å
*b* = 18.5497 (4) Å
*c* = 9.0494 (2) Å
*V* = 1001.22 (5) Å^3^
*Z* = 2
Cu *K*α radiation
μ = 5.32 mm^−1^
*T* = 120 K
0.19 × 0.13 × 0.10 mm

#### Data collection

Oxford Diffraction Xcalibur diffractometer with an Atlas detector
Absorption correction: analytical (*CrysAlis PRO*; Oxford Diffraction, 2009 ▶) *T*_min_ = 0.454, *T*_max_ = 0.678
17493 measured reflections
1749 independent reflections
1739 reflections with *I* > 3σ(*I*)
*R*_int_ = 0.022

#### Refinement

*R*[*F* ^2^ > 2σ(*F* ^2^)] = 0.015
*wR*(*F* ^2^) = 0.049
*S* = 1.21
1749 reflections
126 parameters
1 restraint
H atoms treated by a mixture of independent and constrained refinement
Δρ_max_ = 0.11 e Å^−3^
Δρ_min_ = −0.12 e Å^−3^
Absolute structure: Flack (1983 ▶), 670 Friedel pairs
Flack parameter: 0.008 (20)

### 

Data collection: *CrysAlis CCD* (Oxford Diffraction, 2009 ▶); cell refinement: *CrysAlis RED* (Oxford Diffraction, 2009 ▶); data reduction: *CrysAlis RED*; program(s) used to solve structure: *SIR2002* (Burla *et al.*, 2003 ▶); program(s) used to refine structure: *JANA2006* (Petříček *et al.*, 2006 ▶); molecular graphics: *DIAMOND* (Brandenburg & Putz, 2005 ▶); software used to prepare material for publication: *JANA2006*.

## Supplementary Material

Crystal structure: contains datablocks global, I. DOI: 10.1107/S1600536811006489/ng5118sup1.cif

Structure factors: contains datablocks I. DOI: 10.1107/S1600536811006489/ng5118Isup2.hkl

Additional supplementary materials:  crystallographic information; 3D view; checkCIF report

## Figures and Tables

**Table 1:** Hydrogen-bond geometry (Å, °) *Cg*2 is the centroid of the C3–C8 ring.

*D*—H⋯*A*	*D*—H	H⋯*A*	*D*⋯*A*	*D*—H⋯*A*
O1—H1*o*⋯N1	0.833 (19)	1.905 (18)	2.6506 (13)	148.3 (19)
C1—H1*a*⋯O1^i^	0.96	2.57	3.3351 (11)	137
C8—H8⋯*Cg*2^ii^	0.96	2.85	3.5407 (16)	130
C11—H11*b*⋯*Cg*2^iii^	0.96	2.86	3.728 (2)	150

Symmetry codes: (i) 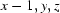; (ii) 
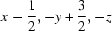
; (iii) 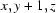.

**Fig. 1.**
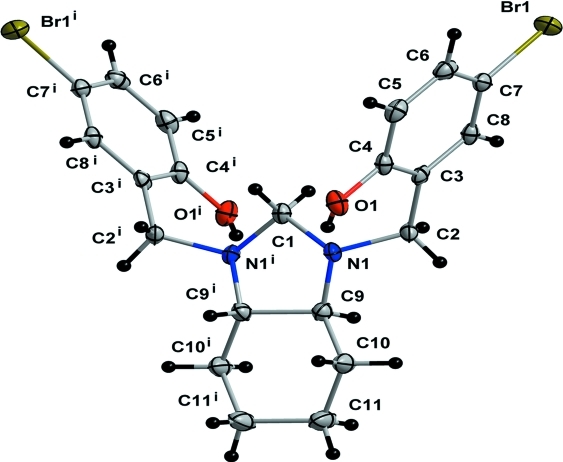
Ellipsoid plot of the title compound. Displacement ellipsoids are drawn at 50% probability level. [Symmetry codes: (i) 1-x, 1-y, z.]

**Fig. 2.**
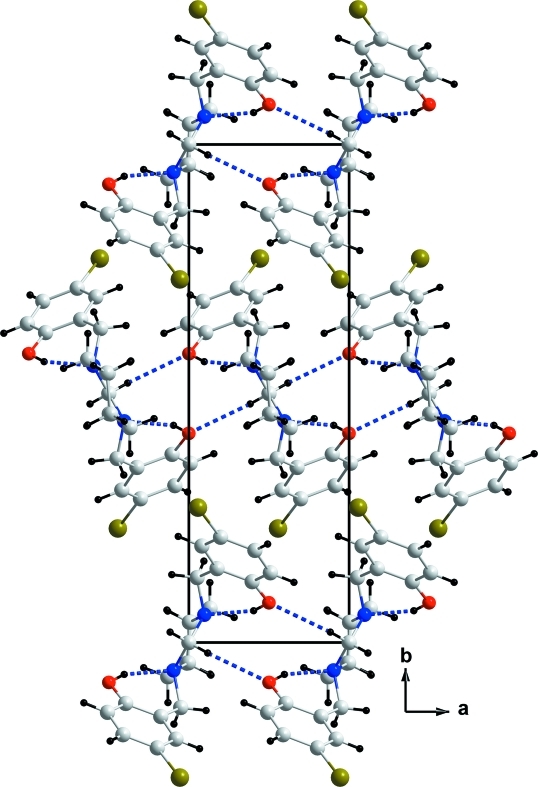
Hydrogen bonding of the molecules of the title compound viewed along c.

## supplementary crystallographic information

### Comment

The title compound was obtained by reaction of racemic cyclic aminal (2*R*,7*R*,11*S*,16*S*)-1,8,10,17-tetraazapentacyclo[8.8.1.1^8,17^.0^2,7^.0^11,16^]icosane with *p*-bromophenol. The molecular structure and atom-numbering scheme for (I) are shown in Fig. 1. Selected angles and bond lengths are listed in Table 1. Its X-ray structure confirms the presence of intramolecular hydrogen bonds between the phenolic hydroxyl groups and nitrogen atoms [N—H, 1.903 (18) Å] that is however by 0.13 Å longer in comparison with related structure (Rivera *et al.*, 2010), whereas the N···O distance [2.6506 (14) Å] is in good agreement with the one found in related structure [2.652 (2) Å]. Similar to this related structure, the observed C—O bond length [1.353 (2) Å] is considerably shortened in relation to other Mannich bases.

In the title compound, C_21_H_24_Br_2_N_2_O_2_, the asymmetric unit contains one-half of the molecule, which is related to the other half by a twofold rotation axis [symmetry code: - *x*, *y*, -*z*] passing through C1. The orthorhombic unit cell contains two molecules. Instead, the molecules have identical (S,*S*)-molecular configuration and its absolute structure was determined with a Flack parameter of 0.01 (2) (Flack, 1983).

### Experimental

*Physical Measurements*

The melting point was determined with an Electrothermal apparatus, and it has not been corrected. IR spectrum was recorded as KBr pellets at 292 K on a Perkin-Elmer Paragon FT—IR instrument. NMR spectra were performed in CDCl_3_ at room temperature on a Bruker AMX 400 Advance spectrometer.

*Preparation of 4,4'-Dibromo-2,2'-(3aS,7aS)-2,3,3a,4,5*,*6,7,7a-octahydro-1H-1,3- benzimidazole-1,3-diyl)bis(methylene)]diphenol) (I)*

A solution of (2*R*,7*R*,11*S*,16S)-1,8,10,17-tetraazapentacyclo[8.8.1.^18,17^.0^2,7^.0^11,16^]icosane (3) (276 mg, 1.00 mmol) in dioxane (3 ml) and water (4 ml), prepared beforehand following previously described procedures, was added dropwise in a dioxane solution (3 ml) containing two equivalents of *p*-bromophenol (346 mg, 2.00 mmol) in a two-necked round-bottomed flask. The mixture was refluxed for about 6 h until precipitation of a colourless solid. The resulting solid was collected by filtration, washed with cool methanol and dried under vacuum (yield 47%, m.p. = 497–499 K). Next, the crude product (100 mg, 0.202 mmol) was dissolved in 5 ml of a 4:1 mixture of chloroform: methanol. Single crystals of title compound (I) suitable for X-ray analysis were grown by slow evaporation of the solvent at room temperature over a period of about 2 weeks in a preferential crystallization (yield 35%). ^1^H NMR (CDCl_3_, 400 MHz): δ 1.27 (4*H*, m), 1.85 (2*H*, m), 2.05 (2*H*, m), 2.33 (2*H*, m), 3.41 (2*H*, d, J = 14.0 Hz, ArCH_2_N), 3.51 (2*H*, s, NCH_2_N), 4.14 (2*H*, d, J = 14.0 Hz, ArCH_2_N), 6.69 (2*H*, d, J = 8.8 Hz), 7.06 (2*H*, s), 7.24 (2*H*, d, J = 8.8 Hz). ^13^C NMR (CDCl_3_, 100 MHz): δ 23.9, 28.9, 55.8, 69.1, 75.7, 111.1, 118.1, 123.4, 130.6, 131.8, 156.6.

### Refinement

All hydrogen atoms were discernible in difference Fourier maps and could be refined to reasonable geometry. According to common practice H atoms attached to C atoms were nevertheless kept in ideal positions during the refinement. The isotropic atomic displacement parameters of hydrogen atoms were evaluated as 1.2**U*_eq_ of the parent atom.

### Crystal data

**Table:**

C_21_H_24_Br_2_N_2_O_2_	*F*(000) = 500
*M**_r_* = 496.2	*D*_x_ = 1.646 Mg m^−^^3^
Orthorhombic, *P*2_1_2_1_2	Cu *K*α radiation, λ = 1.54184 Å
Hall symbol: P 2 2ab	Cell parameters from 16248 reflections
*a* = 5.9645 (2) Å	θ = 4.8–66.5°
*b* = 18.5497 (4) Å	µ = 5.32 mm^−^^1^
*c* = 9.0494 (2) Å	*T* = 120 K
*V* = 1001.22 (5) Å^3^	Prism, colorless
*Z* = 2	0.19 × 0.13 × 0.10 mm

### Data collection

**Table:**

Oxford Diffraction Xcalibur diffractometer with an Atlas detector	1749 independent reflections
Radiation source: X-ray tube	1739 reflections with *I* > 3σ(*I*)
mirror	*R*_int_ = 0.022
Detector resolution: 10.3784 pixels mm^-1^	θ_max_ = 66.8°, θ_min_ = 4.8°
Rotation method data acquisition using ω scans	*h* = −7→6
Absorption correction: analytical (*CrysAlis PRO*; Oxford Diffraction, 2009)	*k* = −10→10
*T*_min_ = 0.454, *T*_max_ = 0.678	*l* = −21→21
17493 measured reflections	

### Refinement

**Table:**

Refinement on *F*^2^	H atoms treated by a mixture of independent and constrained refinement
*R*[*F*^2^ > 2σ(*F*^2^)] = 0.015	Weighting scheme based on measured s.u.'s *w* = 1/[σ^2^(*I*) + 0.0016*I*^2^]
*wR*(*F*^2^) = 0.049	(Δ/σ)_max_ = 0.001
*S* = 1.21	Δρ_max_ = 0.11 e Å^−^^3^
1749 reflections	Δρ_min_ = −0.12 e Å^−^^3^
126 parameters	Absolute structure: Flack (1983), 670 Friedel pairs
1 restraint	Flack parameter: 0.008 (20)
45 constraints	

### Special details

**Table:**

Refinement. The refinement was carried out against all reflections. The conventional *R*-factor is always based on *F*. The goodness of fit as well as the weighted *R*-factor are based on *F* and *F*^2^ for refinement carried out on *F* and *F*^2^, respectively. The threshold expression is used only for calculating *R*-factors *etc*. and it is not relevant to the choice of reflections for refinement.The program used for refinement, Jana2006, uses the weighting scheme based on the experimental expectations, see _refine_ls_weighting_details, that does not force *S* to be one. Therefore the values of *S* are usually larger than the ones from the *SHELX* program.

### Fractional atomic coordinates and isotropic or equivalent isotropic displacement parameters (Å^2^)

**Table:**

	*x*	*y*	*z*	*U*_iso_*/*U*_eq_	
Br1	0.55638 (3)	0.229059 (8)	0.146172 (18)	0.02610 (7)	
O1	0.99958 (19)	0.42005 (6)	0.59844 (13)	0.0226 (3)	
N1	0.59652 (10)	0.44416 (5)	0.71146 (11)	0.0163 (4)	
C1	0.5	0.5	0.61175 (12)	0.0154 (6)	
C2	0.5511 (3)	0.36959 (8)	0.66196 (17)	0.0165 (4)	
C3	0.6757 (3)	0.35153 (7)	0.52184 (17)	0.0156 (4)	
C4	0.8947 (3)	0.37777 (8)	0.49815 (18)	0.0174 (4)	
C5	1.0085 (3)	0.35992 (9)	0.36866 (19)	0.0204 (4)	
C6	0.9120 (3)	0.31499 (8)	0.26478 (18)	0.0205 (5)	
C7	0.6975 (3)	0.28860 (8)	0.29074 (18)	0.0180 (4)	
C8	0.5810 (2)	0.30615 (8)	0.41680 (17)	0.0160 (4)	
C9	0.5029 (3)	0.45921 (8)	0.85825 (17)	0.0179 (4)	
C10	0.6319 (3)	0.43125 (9)	0.99165 (18)	0.0268 (5)	
C11	0.5129 (4)	0.45880 (10)	1.13117 (19)	0.0322 (6)	
H1a	0.382527	0.479008	0.553376	0.0185*	
H2a	0.39302	0.363598	0.646178	0.0198*	
H2b	0.593539	0.336399	0.738387	0.0198*	
H5	1.155662	0.379087	0.351434	0.0245*	
H6	0.991627	0.302257	0.176355	0.0245*	
H8	0.433442	0.286947	0.43234	0.0192*	
H9	0.362416	0.434666	0.870296	0.0214*	
H10a	0.782412	0.449589	0.989185	0.0322*	
H10b	0.63082	0.3795	0.991145	0.0322*	
H11a	0.5957	0.444143	1.217041	0.0386*	
H11b	0.367722	0.43676	1.138666	0.0386*	
H1o	0.904 (3)	0.4361 (12)	0.657 (2)	0.0272*	

### Atomic displacement parameters (Å^2^)

**Table:**

	*U*^11^	*U*^22^	*U*^33^	*U*^12^	*U*^13^	*U*^23^
Br1	0.03375 (13)	0.02617 (12)	0.01839 (12)	−0.00261 (7)	−0.00326 (7)	−0.00361 (7)
O1	0.0184 (6)	0.0183 (5)	0.0312 (7)	−0.0015 (4)	−0.0002 (5)	−0.0041 (5)
N1	0.0203 (7)	0.0104 (6)	0.0182 (7)	0.0013 (5)	0.0010 (5)	−0.0010 (5)
C1	0.0145 (10)	0.0119 (9)	0.0197 (10)	0.0006 (7)	0	0
C2	0.0189 (7)	0.0107 (7)	0.0199 (7)	−0.0017 (5)	0.0040 (7)	−0.0010 (6)
C3	0.0185 (7)	0.0095 (6)	0.0188 (7)	0.0034 (6)	0.0013 (6)	0.0036 (6)
C4	0.0176 (7)	0.0110 (7)	0.0236 (8)	0.0019 (5)	−0.0012 (6)	0.0012 (6)
C5	0.0174 (7)	0.0167 (7)	0.0271 (8)	0.0016 (6)	0.0032 (6)	0.0049 (7)
C6	0.0229 (9)	0.0184 (7)	0.0201 (8)	0.0063 (6)	0.0029 (6)	0.0046 (6)
C7	0.0243 (8)	0.0134 (6)	0.0165 (8)	0.0034 (6)	−0.0013 (6)	0.0005 (6)
C8	0.0143 (7)	0.0119 (7)	0.0220 (8)	0.0018 (5)	−0.0021 (6)	0.0023 (6)
C9	0.0215 (8)	0.0153 (8)	0.0169 (7)	0.0023 (5)	0.0009 (6)	0.0022 (6)
C10	0.0437 (10)	0.0176 (7)	0.0191 (8)	0.0094 (6)	−0.0006 (8)	0.0008 (7)
C11	0.0521 (12)	0.0251 (10)	0.0193 (8)	0.0110 (8)	0.0014 (9)	0.0042 (7)

### Geometric parameters (Å, °)

**Table:**

Br1—C7	1.9080 (16)	C5—H5	0.96
O1—C4	1.353 (2)	C6—C7	1.390 (2)
O1—H1o	0.84 (2)	C6—H6	0.96
N1—C1	1.4895 (12)	C7—C8	1.375 (2)
N1—C2	1.4789 (18)	C8—H8	0.96
N1—C9	1.4677 (18)	C9—C9^i^	1.514 (2)
C1—H1a	0.96	C9—C10	1.523 (2)
C1—H1a^i^	0.96	C9—H9	0.96
C2—C3	1.507 (2)	C10—C11	1.536 (3)
C2—H2a	0.96	C10—H10a	0.96
C2—H2b	0.96	C10—H10b	0.96
C3—C4	1.410 (2)	C11—C11^i^	1.536 (3)
C3—C8	1.390 (2)	C11—H11a	0.96
C4—C5	1.394 (2)	C11—H11b	0.96
C5—C6	1.382 (2)		
			
C4—O1—H1o	108.6 (14)	C5—C6—H6	120.6356
C1—N1—C2	113.34 (9)	C7—C6—H6	120.636
C1—N1—C9	105.60 (9)	Br1—C7—C6	119.62 (12)
C2—N1—C9	112.48 (10)	Br1—C7—C8	118.88 (12)
N1—C1—N1^i^	105.43 (9)	C6—C7—C8	121.48 (15)
N1—C1—H1a	109.4713	C3—C8—C7	120.37 (14)
N1—C1—H1a^i^	109.4712	C3—C8—H8	119.8142
N1^i^—C1—H1a	109.4712	C7—C8—H8	119.8142
N1^i^—C1—H1a^i^	109.4713	N1—C9—C9^i^	101.48 (12)
H1a—C1—H1a^i^	113.2317	N1—C9—C10	117.42 (13)
N1—C2—C3	111.86 (12)	N1—C9—H9	110.1556
N1—C2—H2a	109.4713	C9^i^—C9—C10	110.62 (13)
N1—C2—H2b	109.4715	C9^i^—C9—H9	117.0073
C3—C2—H2a	109.471	C10—C9—H9	100.9265
C3—C2—H2b	109.4709	C9—C10—C11	107.74 (15)
H2a—C2—H2b	106.9725	C9—C10—H10a	109.471
C2—C3—C4	120.52 (13)	C9—C10—H10b	109.4714
C2—C3—C8	120.64 (14)	C11—C10—H10a	109.4709
C4—C3—C8	118.78 (14)	C11—C10—H10b	109.4716
O1—C4—C3	121.76 (14)	H10a—C10—H10b	111.1472
O1—C4—C5	118.47 (13)	C10—C11—C11^i^	112.17 (15)
C3—C4—C5	119.78 (14)	C10—C11—H11a	109.4713
C4—C5—C6	120.83 (14)	C10—C11—H11b	109.4711
C4—C5—H5	119.5819	C11^i^—C11—H11a	109.4712
C6—C5—H5	119.5833	C11^i^—C11—H11b	109.4713
C5—C6—C7	118.73 (15)	H11a—C11—H11b	106.632
			
C2—N1—C1—N1^i^	138.00 (9)	O1—C4—C5—C6	177.87 (15)
C9—N1—C1—N1^i^	14.44 (9)	C3—C4—C5—C6	−1.9 (2)
C1—N1—C2—C3	68.53 (14)	C4—C5—C6—C7	0.9 (2)
C9—N1—C2—C3	−171.77 (12)	C5—C6—C7—Br1	177.93 (12)
C1—N1—C9—C10	−157.29 (11)	C5—C6—C7—C8	−0.1 (2)
C1—N1—C9—C9^i^	−36.62 (13)	Br1—C7—C8—C3	−177.66 (11)
C2—N1—C9—C10	78.61 (16)	C6—C7—C8—C3	0.4 (2)
C2—N1—C9—C9^i^	−160.72 (12)	N1—C9—C10—C11	175.39 (13)
N1—C2—C3—C4	37.37 (19)	C9^i^—C9—C10—C11	59.64 (18)
N1—C2—C3—C8	−145.54 (13)	N1—C9—C9^i^—N1^i^	45.12 (14)
C2—C3—C4—O1	−0.5 (2)	N1—C9—C9^i^—C10^i^	170.46 (12)
C2—C3—C4—C5	179.31 (14)	C10—C9—C9^i^—N1^i^	170.46 (12)
C8—C3—C4—O1	−177.62 (14)	C10—C9—C9^i^—C10^i^	−64.21 (18)
C8—C3—C4—C5	2.2 (2)	C9—C10—C11—C11^i^	−55.1 (2)
C2—C3—C8—C7	−178.55 (14)	C10—C11—C11^i^—C10^i^	54.8 (2)
C4—C3—C8—C7	−1.4 (2)		

Symmetry codes: (i) −*x*+1, −*y*+1, *z*.

### Hydrogen-bond geometry (Å, °)

**Table:**

Cg2 is the centroid of the C3–C8 ring.

**Table:**

*D*—H···*A*	*D*—H	H···*A*	*D*···*A*	*D*—H···*A*
O1—H1o···N1	0.833 (19)	1.905 (18)	2.6506 (13)	148.3 (19)
C1—H1a···O1^ii^	0.96	2.57	3.3351 (11)	137
C8—H8···Cg2^iii^	0.96	2.85	3.5407 (16)	130
C11—H11b···Cg2^iv^	0.96	2.86	3.728 (2)	150

Symmetry codes: (ii) *x*−1, *y*, *z*; (iii) *x*−1/2, −*y*+3/2, −*z*; (iv) *x*, *y*+1, *z*.
